# Supplementary material for: Western US intraplate deformation controlled by the complex lithospheric structure
Source: Nat Commun. 2024 May 9;15:3917. doi: 10.1038/s41467-024-48223-2 (PMC11082152; doi:10.1038/s41467-024-48223-2)
Supplement: Supplementary file 1 — Supplementary Information [file 41467_2024_48223_MOESM1_ESM.pdf]

Western US Intraplate Deformation Controlled by the Complex Lithospheric Structure

Zebin Cao[1, 2], Lijun Liu[1, 2*]

1. State Key Laboratory of Lithospheric Evolution, Institute of Geology and Geophysics, Chinese Academy of Sciences, Beijing, China.
2. Department of Earth Science & Environmental Change, University of Illinois at Urbana-Champaign, IL, USA.

Correspondence to: *ljliu@mail.iggcas.ac.cn

This file includes:

Supplementary Figures 1 to 7

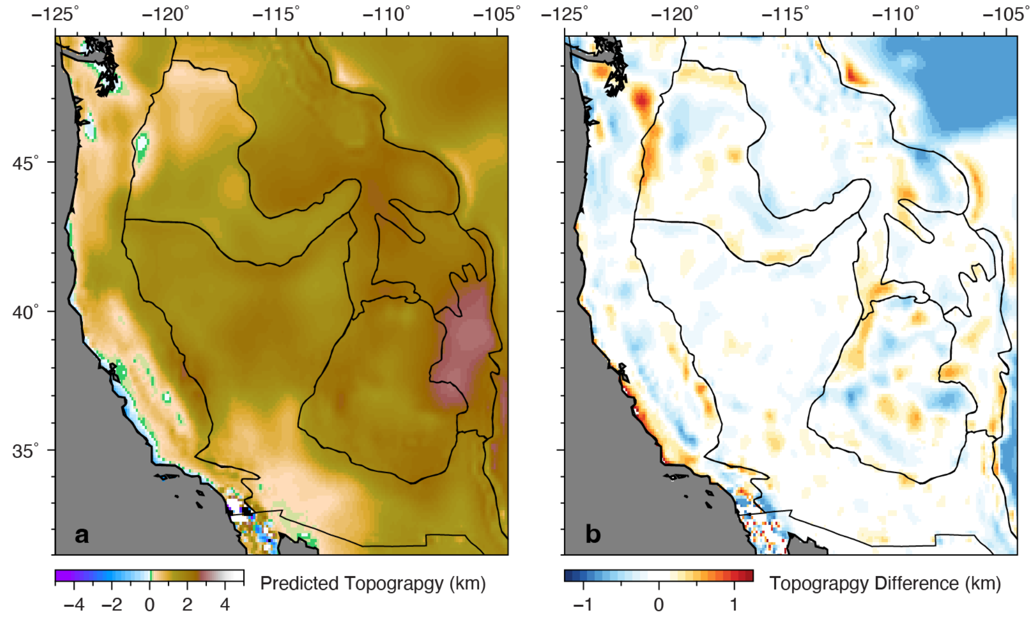

Supplementary Figure 1. Predicted topography. (**a**) Topography predicted by the best-fitting model, M3. (**b**) Difference between the observed and predicted topography. A negative value means the prediction overestimates the topography.

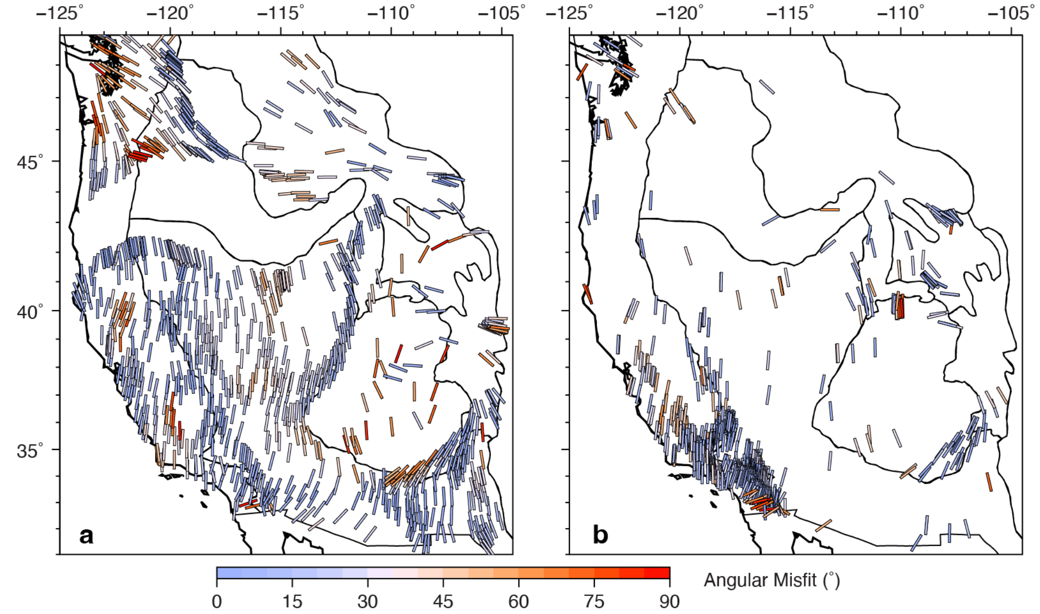

Supplementary Figure 2. Comparisons to S_{Hmax} observations. The bars show the predicted orientations from the best-fitting model, M3, and the colors represent angular misfits relative to that observed, with the dataset from (**a**) Levandowski et al.[25] and (**b**) Lund Snee and Zoback[26].

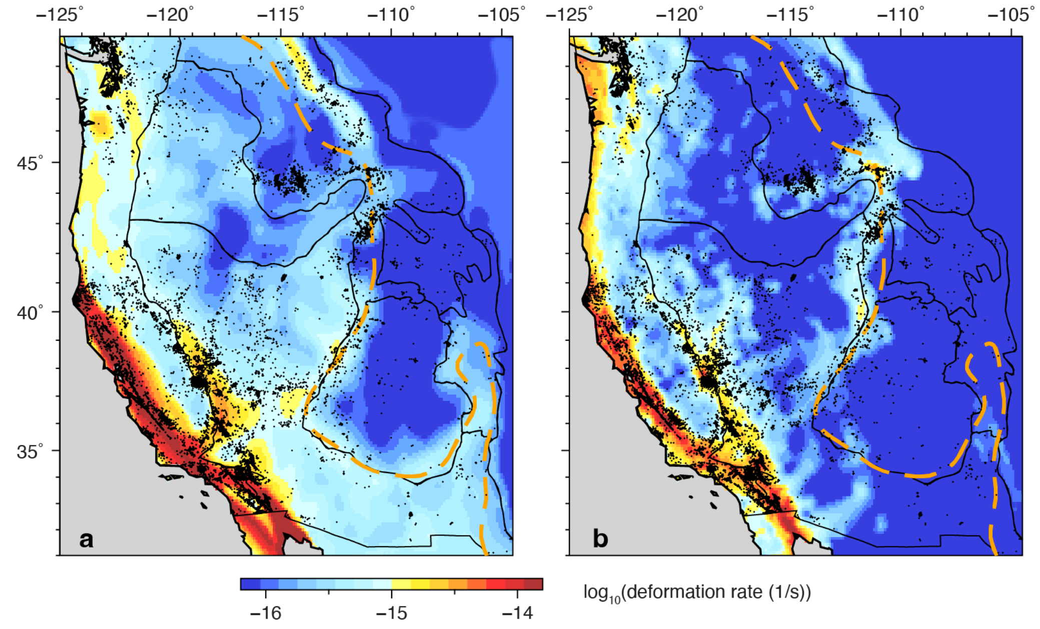

Supplementary Figure 3. Predicted and observed surface deformation rate field. (**a**) Predicted depth-averaged deformation rate in the uppermost 10 km from the best-fitting model, M3, and (**b**) observed surface deformation rate[43]. The black dots indicate earthquake locations from 1980 to 2020 (from USGS). The orange dashed lines mark the location of LAB at 75 km, approximating the lithospheric thickness step in LTM1.

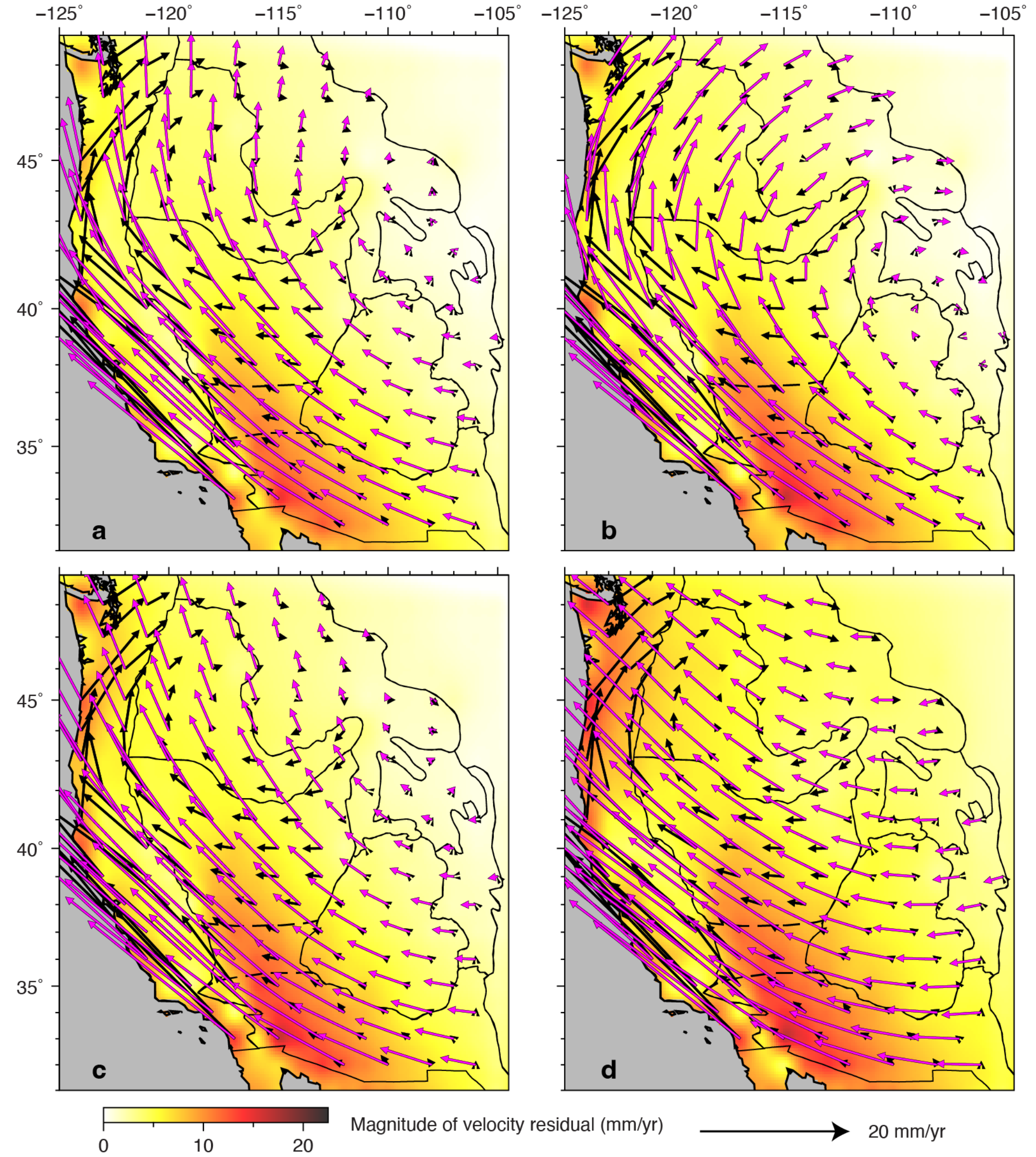

Supplementary Figure 4. Predicted WUS crustal motion with a flat LAB and different driving forces. The predicted crustal motion with (**a**) plate boundary forces only, (**b**) plate boundary forces and basal tractions, (**c**) plate boundary forces and crustal GPE generated by a uniform crust with 30-km constant Moho depth, and (**d**) plate boundary forces and crustal GPE generated by a crust with laterally varying Moho depth.

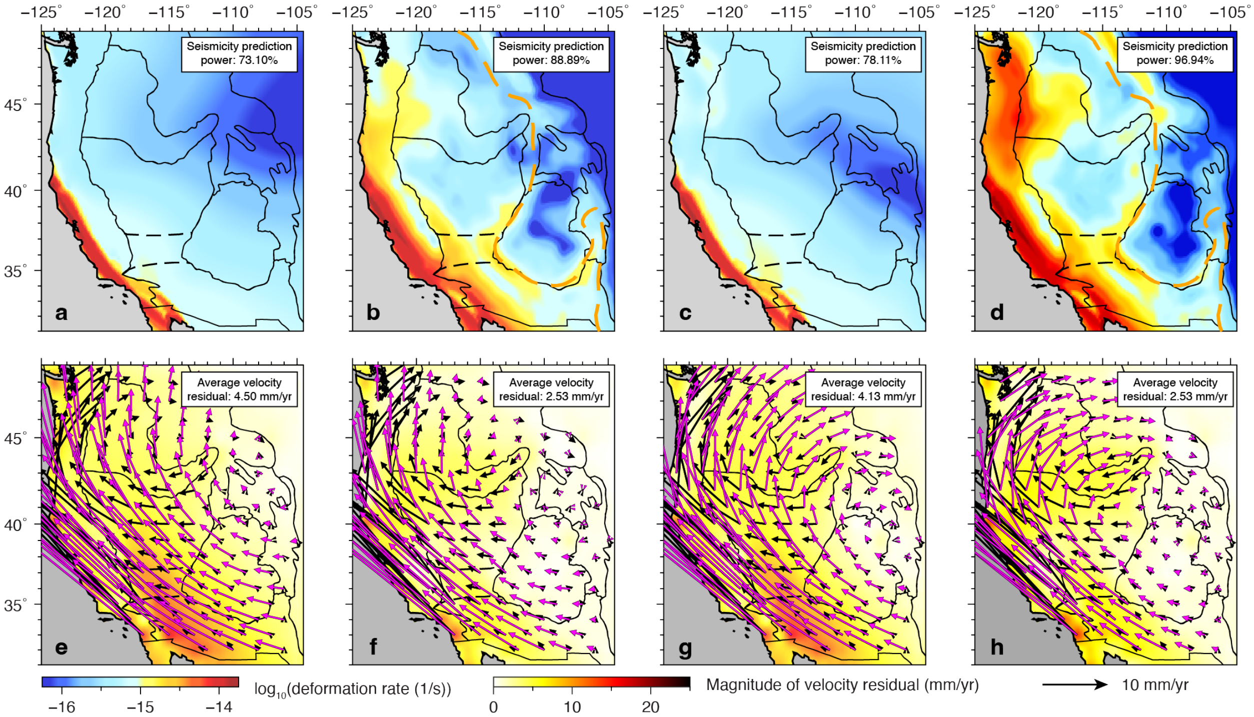

Supplementary Figure 5. Predicted crustal deformation due to mantle convection from models with different lithospheric and convective mantle structures. Predicted crustal deformation rate in the uppermost 30 km from (**a**) M7 with LTM2, a uniform lithospheric effective viscosity structure, and a none convective mantle, (**b**) M8 with LTM1, a 3D lithospheric effective viscosity structure, and a none convective mantle, (**c**) M9 with a lithosphere the same as M7 and all density anomalies in the convective mantle, and (**d**) M10 with a lithosphere the same as M8 and all density anomalies in the convective mantle. Comparisons between the GPS-measured surface motion and predicted surface motion from (**e**) M7, (**f**) M8, (**g**) M9, and (**h**) M10. In (e) – (h), the black bars represent the smoothed GPS measurements, and the magenta bars represent the predictions.

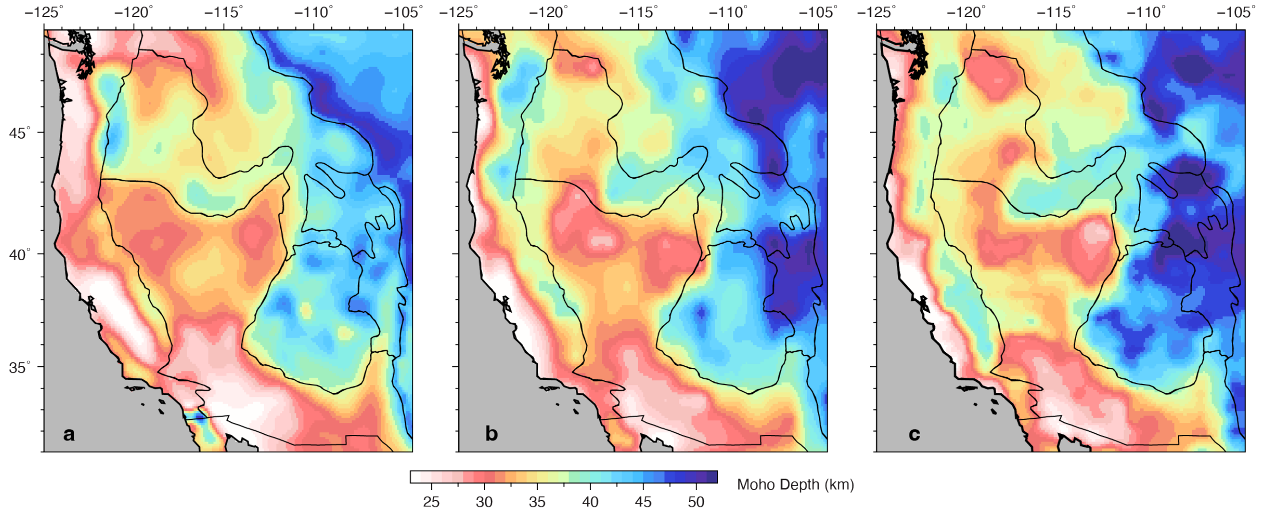

Supplementary Figure 6. Moho depth models. (**a**) The Moho depth model used in the geophysically inferred 3D lithospheric structure (Methods), (**b**) Moho depth from CRUST 1.0[49], and (**c**) Moho depth from Schmandt et al.[78]

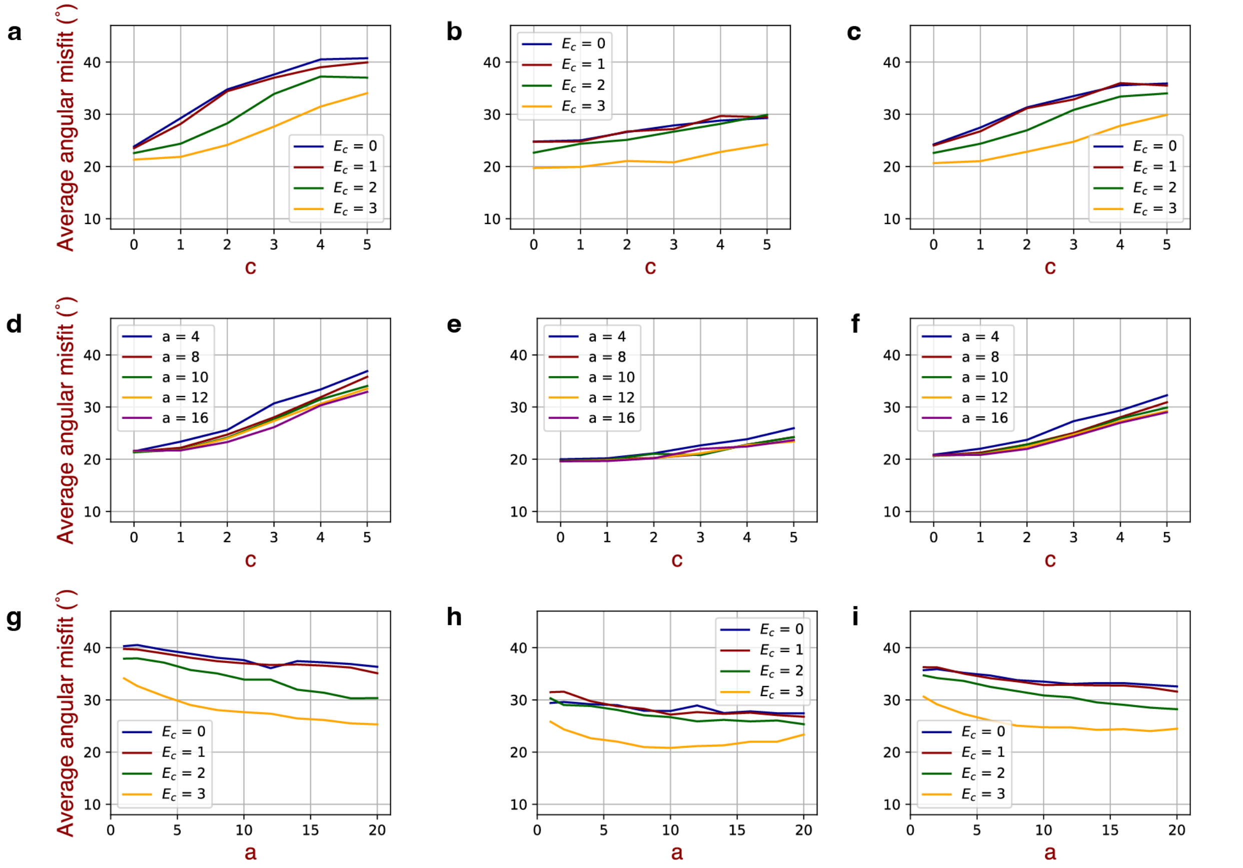

Supplementary Figure 7. Results of parameter search for 3D crustal effective viscosity structure. (**a**) – (**c**) Comparisons between model prediction and observation with a fixed a = 10 and varying E_c and c. (**d**) – (**f**) Comparisons between model prediction and observation with a fixed E_c = 3 and varying a and c. (**g**) – (**i**) Comparisons between model prediction and observation with a fixed c = 3 and varying E_c and a. (a), (d), and (g) show the average angular misfit of S_{Hmax} directions compared to Levandowski et al.[25] (b), (e), and (h) show the average angular misfit of S_{Hmax} directions compared to Lund Snee and Zoback[26]. (c), (f), and (i) show the average angular misfit of S_{Hmax} directions compared to the two datasets combined.
